# A randomized controlled clinical trial of concentrated growth factor combined with sodium hyaluronate in the treatment of temporomandibular joint osteoarthritis

**DOI:** 10.1186/s12903-024-04258-x

**Published:** 2024-05-08

**Authors:** Xin-yu Jia, Si-li Jing, Yang Sun, Zhong-cheng Gong, Zhi-chen Guo

**Affiliations:** 1 Key Laboratory of Shaanxi Province for Craniofacial Precision Medicine Research, College of Stomatology, Xi’an Jiaotong University, Xi’an, 710004 China; 2 Clinical Research Center of Shaanxi Province for Dental and Maxillofacial Diseases, College of Stomatology, Xi’an Jiaotong University, Xi’an, 710004 China; 3 Department of Oral and Maxillofacial Surgery, College of Stomatology, Xi’an Jiaotong University, No. 98 Xiwu Road, Xi’an, 710004 China; 4 Department of General Dentistry and Emergency Room, College of Stomatology, Xi’an Jiaotong University, Xi’an, 710004 China; 5 Shannxi Eye Hospital, Xi’an People’s Hospital (Xi’an Fourth Hospital), Affiliated People’s Hospital of Northwest University, Xi’an, 710004 China; 6 Department of Oral and Maxillofacial Surgery, The Third Affiliate Air Force Military Medical University, Xi’an, 710004 China; 7 Oncological Department of Oral and Maxillofacial Surgery, The Affiliated Stomatology Hospital of The First Affiliated Hospital of Xinjiang Medical University, No. 137, Li Yu Shan South Road, Urumqi, 830054 Xinjiang China

**Keywords:** Concentrated growth factor, Sodium hyaluronate, Temporomandibular joint, Temporomandibular joint osteoarthrosis, Condylar repair

## Abstract

**Objective:**

To investigate the effect of concentrated growth factor (CGF) combined with sodium hyaluronate (SH) on temporomandibular joint osteoarthritis (TMJOA).

**Methods:**

Sixty patients with TMJOA who were diagnosed by cone-beam computed tomography (CBCT) between March 2020 and March 2023 at the Stomatological Hospital of Xi’an Jiaotong University were randomly divided into a control group (*n* = 30) and an experimental group (*n* = 30). The patients in the experimental group were treated with CGF + SH, and those in the control group were treated with SH only. The visual analogue scale (VAS) score indicating pain in the temporomandibular joint (TMJ) area; the Helkimo Clinical Dysfunction Index (Di); and changes in condylar CBCT at the first visit and 2 weeks, 3 months and 6 months after treatment were recorded. The CBCT data of the patients in the experimental and control groups were collected, and the three-dimensional CBCT image sequences were imported into Mimics Medical 19.0 software in DICOM format for condylar reconstruction.

**Results:**

The VAS scores at 2 weeks, 3 months and 6 months after treatment were significantly lower in the experimental group than in the control group (*P* < 0.05), and the pain in the experimental group was significantly relieved. The Di was significantly lower in the experimental group than in the control group (*P* < 0.05), and the clinical function of the TMJ improved. After treatment, the CBCT score was significantly lower in the experimental group than in the control group (*P* < 0.05), and the condylar bone cortex was obviously repaired. Observation of the condylar bone cortex by three-dimensional reconstruction showed the same results as those obtained by CBCT.

**Conclusion:**

CGF combined with SH is effective in the treatment of TMJOA and can improve muscle pain, TMJ pain, Impaired TMJ function, Impaired range of movement, Pain on movement of the mandible and promote bone repair.

**The registration number (TRN):**

ChiCTR2400082712.

**The date of registration:**

April 5, 2024.

**Supplementary Information:**

The online version contains supplementary material available at 10.1186/s12903-024-04258-x.

## Introduction

Temporomandibular joint osteoarthritis (TMJOA) is a chronic degenerative disease that occurs in the TMJ area and is caused by many factors. It is the most serious type of TMJ disorder [1] and usually leads to destruction of the mandibular condyle and articular fossa due to an increase in joint load. TMJOA can cause varying degrees of clinical symptoms, including joint pain, clicking, limited movement and limited mouth opening, resulting in loss of joint function [2]. At present, conservative treatments, most commonly intra-articular injections, are the main treatment strategies applied in the clinic. Many kinds of drugs, including hormones, ozone and sodium hyaluronate (SH), are used in these treatments. Hormones can relieve pain and increase the range of motion of joints in the short term, but they can also cause damage to the cartilage and bone structure in the articular cavity. Ozone causes strong oxidation, which can promote cell metabolism and enhance cell repair, but these effects persist for a short time [3]. These treatments can improve the symptoms of TMJOA, but there is no gold standard for treating this disease [4–9].

Sodium hyaluronate (SH) is the main component of synovial fluid and can reduce friction caused by joint motion, lubricate joints, improve physiological joint function, and protect joints through anti-inflammatory mechanisms [10, 11]. SH is the most commonly used drug for intra-articular injections in the treatment of TMJOA. The injection of sodium hyaluronate into the upper cavity of the temporomandibular joint is more effective than into the subarticular cavity for the treatment of synovitis of the joint. A possible reason is that, compared with that of the subarticular cavity, the volume of the upper cavity is larger, and the injection is relatively easy [12]. However, it has been shown that the subarticular cavity surface is rougher than the upper cavity surface and is often prone to condylar surface fibrosis and degeneration. Subarticular cavity injection can significantly improve the clinical outcome in TMD patients, but the effect of upper cavity injection is poor [13]. SH has been widely used in the TMJ and other large joints. For example, the clinical efficacy and safety of intra-articular injections of SH in the knee joint, ankle joint and hip joint have been systematically reviewed and supported by clinical studies, but most of the previously published articles have focused on changes in clinical signs and symptoms after intra-articular injections of SH [13–16]. There are no clear results showing that SH can mitigate bone destruction.

Platelet-rich plasma (PRP), which comes from autologous sources, rarely results in an immune rejection reaction. Regimens of SH combined with PRP have been used in the clinical treatment of TMJOA [17, 18], but PRP carries some potential immunogenic risk and has a short action time. Additionally, its effect on hard tissue recovery has not been determined. Thus, new replacements are urgently needed in the clinic. Autologous concentrated growth factor (CGF) contains more growth factors; has a stronger ability to support tissue regeneration and repair; can better promote the regeneration of bone, blood vessels, fibres and other tissues; and has greater biosafety. CGFs can be used to reconstruct damaged bone and cartilage, achieve intra-articular homeostasis, and regulate inflammation [19]. At present, CGF has been used to treat OA in all parts of the body, but there have been few reports on the application of CGF in the TMJ. In the present study, patients were randomly divided into groups according to the use of CGF as an endogenous growth factor source to analyse the advantages of CGF combined with SH in the treatment of TMJOA. Through various scoring standards, this work proposes a new theoretical basis for the clinical treatment of TMJOA.

## Materials and methods

### General information of the TMJOA patients

**Fig. 1:**
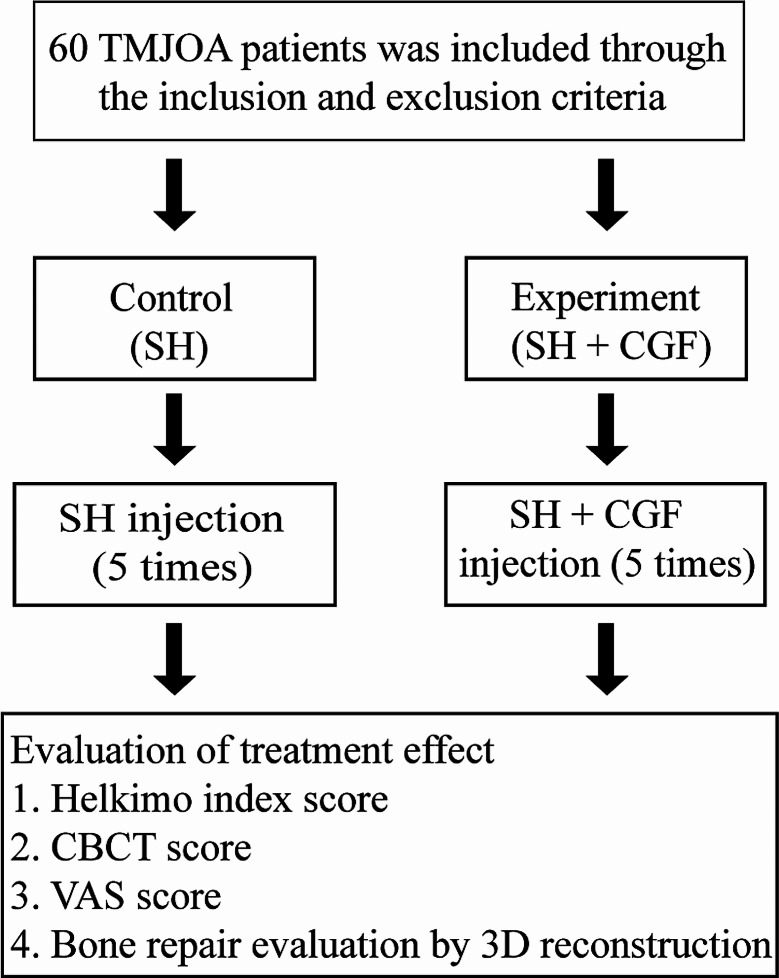
Flow diagram of the experimental design

Sixty-two TMJOA patients were recruited from the Department of Oral and Maxillofacial Surgery, College of Stomatology, Xi’an Jiaotong University, between March 2020 and March 2023 (in this study, all 62 patients with TMJOA had unilateral joint involvement). The project was approved by the ethics review board (approval no. xjkqll[2018]NO.021). This experiment followed the consolidated Standards of Reporting Trials (COSORT) reporting guidelines. The flow diagram is shown in Fig. 1. The inclusion criteria were as follows: (1) had a diagnosis of TMJOA according to patient history, clinical symptoms, joint function and imaging examination and treatment by the same physician at the Stomatology Hospital of Xi’an Jiaotong University, and the injection was performed by the same doctor; (2) voluntarily agreed to participate in the study and provided signed informed consent; and (3) were in good overall condition without serious systemic diseases or mental health conditions. The exclusion criteria were as follows: (1) a history of injections of other drugs or invasive procedures in the joint area within 1 month; (2) severe bruxism and obvious malocclusion; (3) rheumatoid disease; (4) a history of severe pulmonary or heart disease; (5) diabetes or poor blood glucose control; (6) pregnancy or lactation; (7) incomplete data; and (8) use of blood products or antithrombotic drugs within 2 weeks before blood centrifugation. A total of 62 patients agreed to participate in clinical studies when they were treated for TMJOA. All patients were informed of the treatment in the trial. The age of the patients ranged from 18 to 50 years. Two patients were not suitable for this test because of diabetes. Therefore, a total of 60 patients were enrolled in the experiment, including 30 females and 30 males. All patients were divided into two groups (control group: SH; experimental group: SH + CGF). There were 30 target patients in each group. (Table 1).

**Table 1:** Demographic characteristics of included patients

Variable	Control (cases/sides)	Experimental (cases/sides)
Patients with TMJOA	30(30)	30(30)
Age	30.72 ± 7.10	31.78 ± 7.13
Number of patients (Male/Female)	30(20/10)	30(22/8)
Differentiation		
Bone destruction	22	24
Sclerosis	1	3

### Randomization

The control group (SH) and the experimental group (SH + CGF) were written on sheets of paper and enclosed in opaque, sealed envelopes. The envelopes were randomly and equally distributed to patients, and the envelopes were given to doctor A for injection. Doctor B conducted the clinical analysis and collected the data. The grouping results were announced 6 months after treatment.

### Study variables

The primary variable of this clinical study was CGF application. The primary outcome variables were the degree of joint pain and extent of bone repair. The secondary outcome variables were TMJ function (joint sounds, locking or luxation of the joint), impaired range of movement, muscle pain, and pain during movement of the mandible.

### Preparation of SH + CGF

Peripheral blood was collected from four millilitres of TMJOA patients using a special tube for CGF separation. One millilitre of CGF was extracted, and the hybrid of SH and CGF was mixed at a 1:1 ratio.

### Treatment methods

Before the injection, the patient was informed of the treatment plan, and after the injection, the follow-up time was 2 weeks, 3 months, and 6 months. The experiment could be terminated at any time if the patient requested withdrawal or if adverse complications occurred. In the SH treatment group, the patients assumed a sitting position, with 1 cm in front of the tragus and the line between the lateral canthus and the tragus marked by approximately 2 mm. The patient was instructed to open their mouth repeatedly while the doctor touched the condyle on the affected side. The doctor marked the needle entry point outside the condyle and disinfected the operation area with 0.5% povidone iodine with the mouth closed; the needle was inserted vertically with respect to the outer pole of the condyle, and the patient was instructed to open the mouth a small amount. At this time, the needle can move with the condyle; if this occurs, the doctor slides the injection needle tip upwards and back to the posterior slope of the condyle and then injects 1 ml of lidocaine if there is no resistance, withdrawing some fluid to confirm that the needle enters the subarticular cavity. After the joint cavity was repeatedly washed, the flushing fluid was removed, and 1 ml of SH was injected. After the injection, the patient was instructed to open and close the mouth repeatedly to evenly distribute the drug in the joint cavity. All patients were instructed to avoid opening their mouths widely, pay attention to avoiding biting hard objects in their diet, and rest and stay warm. In the experimental group, 0.5 ml of CGF combined with 0.5 ml of SH was injected. In the control group, 1 ml of SH was injected, and the procedure was the same as that in the experimental group.

### Observation indices and judgement methods

Pain was assessed with a visual analogue scale (VAS) [20] before treatment and 2 weeks, 3 months and 6 months after treatment. The degree of pain in the two groups was compared using a 10-cm-long sliding scale with 10 points on one side and “0” and “10” points on the two ends. A score of 10 indicated the most intense pain, and a score of 0 indicated no pain.

The Helkimo Clinical Dysfunction Index (Di) [21] was used to analyse the function of the TMJ before and after treatment to quantitatively evaluate the clinical effect of SH and SH combined with CGF on TMJOA. Patients were given a score of 0 points for the absence of symptoms, 1 point for mild pain or dysfunction, and 5 points for severe pain or dysfunction (Supplementary Table 1).

Evaluation of the TMJ by cone-beam computed tomography (CBCT) was performed to observe changes in the condylar cortical bone before treatment and 3 and 6 months after treatment [22]. CBCT image acquisition: All patients underwent high-resolution CBCT of the TMJ under uniform conditions. A head positioner and cursor positioning system were used to position the midsagittal plane of the face of the patient vertical to the ground and the Frankfurt horizontal plane parallel to the ground. Patients remained immobile in the mandibular postural position (binocular smooth inspect facing forward, no chewing, no swallowing, no speaking, and the upper and lower dentitions naturally maintaining the intercuspal position) during the scanning procedure. The technical parameters were as follows: tube voltage, 85 kilovolt peak; effective tube current, 7 mA; thickness layer of the scanning process, 0.15 mm; reconstructed slice thickness, 0.625 mm; reconstructive interval, 0.5 mm; revolution speed, 1 s/rotation; and matrix, 512 × 512. The CBCT protocol included the LARGEV (Smart3D-X) unit with an FOV of 20 × 18 cm, isotropic voxels of 0.2 mm in axial slice thickness, and a total scan time of 15 s. The CBCT score was determined according to the condition of the condylar lesions. The most important lesions were selected and scored on the coronal and sagittal planes by CBCT. The condylar bone change score was the sum of the coronal and sagittal scores, with the total score ranging from 0 ∽ 6 (the total score for condylar ossification and condylar cystic lesions was 4) (Supplementary Table 2).

Three-dimensional reconstruction of the condyle was performed in the experimental and control groups [23, 24] as follows: (1) A new project was created in DICOM format in Mimics 19 software, and the direction of the image was determined in the direction window. (2) The bone scale (BS) was selected from the list of greyscale values in the contrast toolbar (grayscale value range: 1024–1650 HU). (3) For the new masks, the segmentation threshold was set to 226 ∽ 3071 HU, and the Edit Masks tool was used to erase the image content except for the condyle layer by layer on the sagittal, coronal and horizontal planes; the integrity of the condyle was also examined. (3) The upper condylar boundary was defined by the first high-density image in the articular fossa observed on the horizontal plane; continuing to move to lower layers, the inferior condylar boundary was defined by the image immediately above the image showing the condylar process connected with the coracoid process. (4) The Calculate 3D mask command was used to reconstruct the unilateral condylar model.

### Statistical analysis

SPSS 22.0 was used for all the statistical analyses. The normality of the data was tested by the Shapiro‒Wilk method. Normally distributed data are expressed as the average ± standard deviation ($\stackrel{-}{x}$±s), and the t test was used for statistical analysis. Nonnormally distributed data are expressed as quartiles (25th percentile, 75th percentile), and the Mann–Whitney U test was used for statistical analysis. The difference was statistically significant (*P* < 0.05).

## Results

### The preparation of SH + CGF

**Fig. 2:**
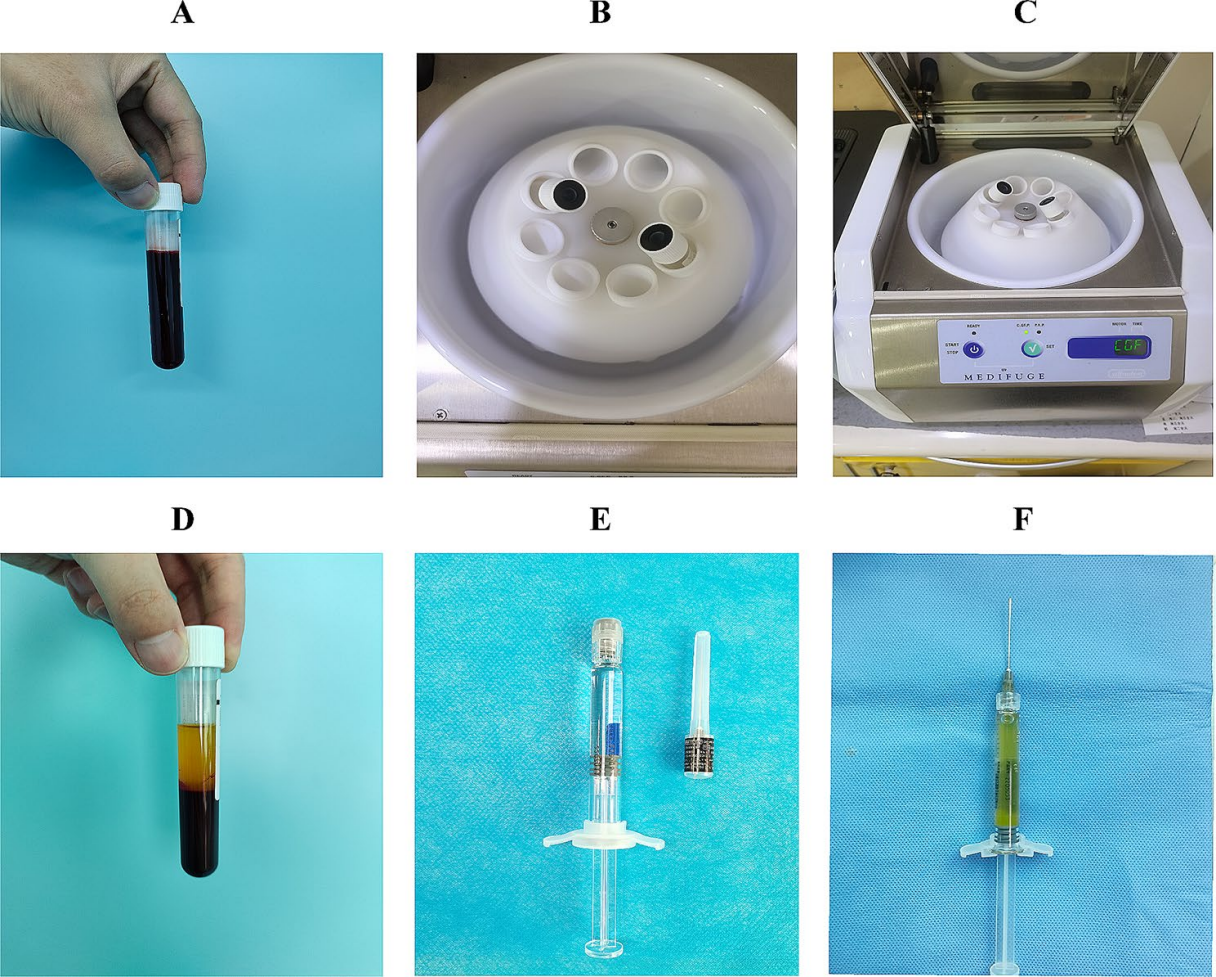
**A**-**C**: CGF was prepared by blood centrifugation. **D**-**F**: Prepare the hybrid of CGF and SH at a 1:1 ratio

Before treatment, the patient’s venous blood was centrifuged to obtain CGF, and approximately 1 ml was extracted; the sample was evenly mixed with SH without obvious solidification. (Fig. 2)

### Pain relief in TMJOA patients treated with SH + CGF

Before treatment, there was no significant difference in the VAS score between the two groups (*P* > 0.05). However, the score of the control group decreased after treatment. However, the VAS scores at 2 weeks, 3 months and 6 months after treatment were significantly lower in the experimental group than in the control group (*P* < 0.05). The results showed that while the degree of pain decreased in both groups after treatment, the degree of pain relief in the experimental group was significantly greater than that in the control group (Table 2).

**Table 2:** Mann-Whitneyu statistical analysis of VAS score in temporomandibular joint region before and after treatment in two groups of patients with temporomandibular joint bone-related diseases. Median(P25, P75)

Group	Number	Before treatment	2 weeks after treatment	3 months after treatment	6 months after treatment
		Median (P25, P75)	Median (P25, P75)	Median (P25, P75)	Median (P25,P75)
Control	30	7.00(6.00,7.00)	5.00 (4.0,5.25)	3.00 (3.00,4.00)	2.00 (2.00,3.00)
Experiment	30	7.00(6.00,7.00)	4.00 (4.00,4.25)	3.00 (2.00,3.00)	1.00 (1.00,2.00)
*P* value		0.861	0.001	0.001	0.000

*Note* The control group is SH treatment group and the experimental group is CGF + SH treatment group. (*P* < 0.05)

### SH + CGF promoted functional recovery of the TMJ in TMJOA patients

There was no significant difference in the Di between the two groups before treatment. The Di indices in the experimental and control groups were significantly lower at 2 weeks, 3 months and 6 months after treatment than before treatment (*P* < 0.001). The Di at 2 weeks, 3 months and 6 months after treatment was significantly lower in the experimental group than in the control group (*P* > 0.05). The results showed that the range of movement of the mandible, joint pain, and muscle pain of the patients in both groups significantly improved after treatment and that the conditions of the patients in the experimental group were better than those of the patients in the control group (Table 3).

**Table 3:** Helkimo index of two groups of patients with bone disease of temporomandibular joint before and after treatment (score, $\stackrel{-}{x}$±s)

Group	Number	Before treatment	2 weeks after treatment	3 months after treatment	6 months after treatment
Control	30	9.07 ± 3.73	7.07 ± 3.13^a^	4.13 ± 1.04^ab^	3.93 ± 2.16^abc^
Experimental	30	8.93 ± 3.82	5.30 ± 2.61^a^	3.17 ± 1.60^ab^	2.70 ± 1.42^abc^
*P* value		0.891	0.021	0.007	0.011

*Note* The control group was SH treatment group and the experimental group was CGF + SH treatment group. a, b and c were compared with the patients in the same group before treatment, 2 weeks after treatment and 2 months after treatment (*P* < 0.05)

### SH + CGF can accelerate condylar bone reconstruction in TMJOA patients

**Fig. 3:**
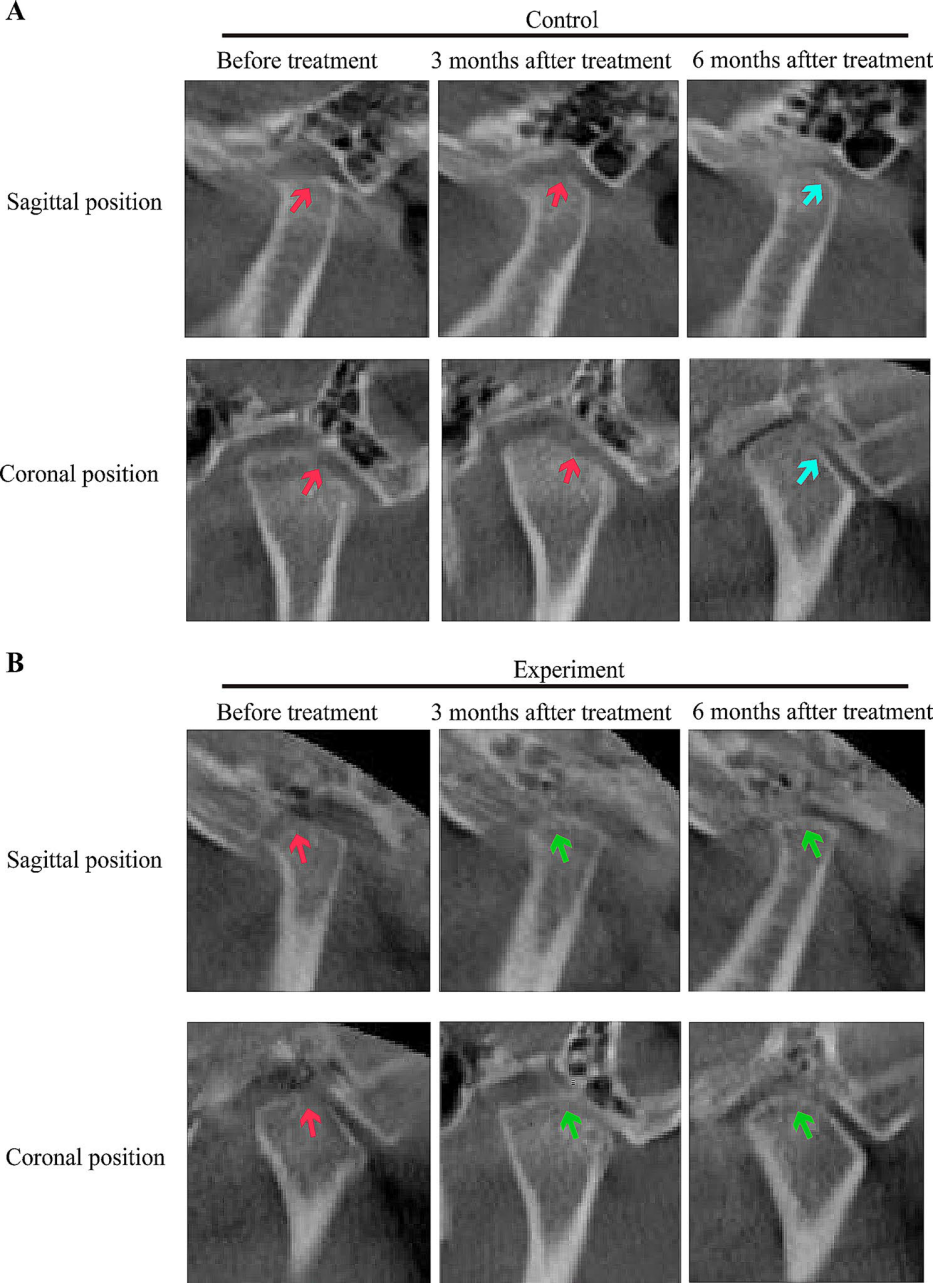
Evaluation of the TMJ bone condition by CBCT. Obvious destruction of the condyle was observed in both groups before treatment (red arrow). No further destruction was observed in the control group at 3 or 6 months after treatment, and partial repair was observed at 6 months (light blue arrow). In the experimental group, obvious repair of the condylar bone was observed on the coronal plane and sagittal plane at 3 months and 6 months after treatment (green arrow)

**Fig. 4:**
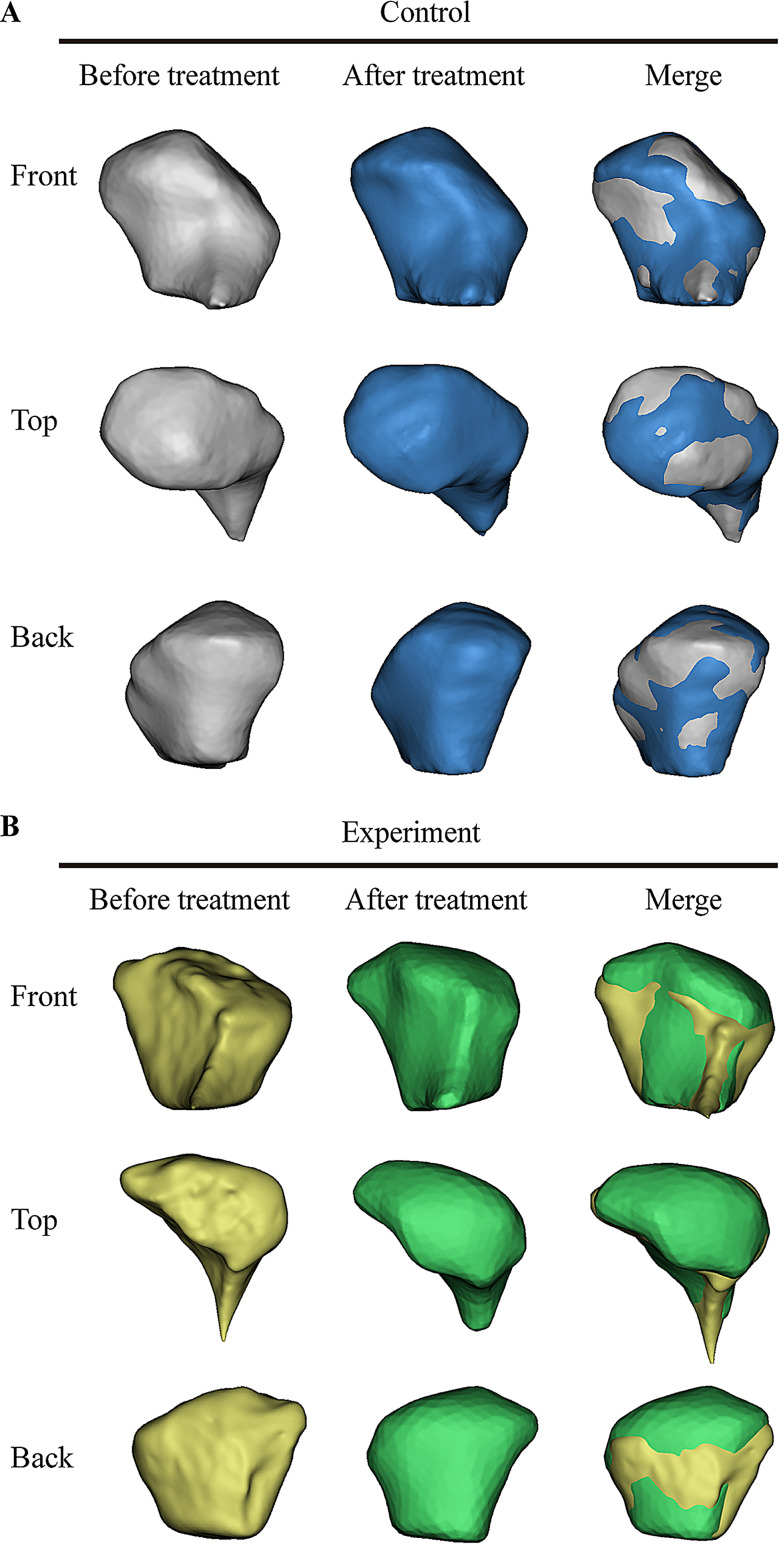
There was no significant change in the control group after treatment (blue) compared with before treatment (grey). After the plants were merged, there were no large areas of colour repetition or obvious bone repair in the control group. The condylar repair area in the experimental group after treatment (green) was larger than that before treatment (yellow), and large areas of colour repetition and condylar repair could be observed after merging

All the condyles of the patients in the experimental and control groups exhibited different degrees of degenerative changes before treatment, such as condylar flattening, wear, osteosclerosis, and osteophyte formation. There was no significant difference in the CBCT score before treatment (*P* > 0.05). Of the 60 TMJOA patients, 77% (46/60) had condylar erosion, 6.7% (4/60) had osteophytes, 6.7% (4/60) had sclerosis, and 10% (6/60) had condylar flattening. With the progression of treatment, the edge line of the condylar surface gradually changes from rough to smooth, and cortical bone gradually forms on the condylar surface. No further destruction of bone was found in any of the patients (Fig. 3). Through three-dimensional reconstruction, we directly observed partial repair of the condyle from the top of the condyle to the slope of the outer pole after treatment in the control group, but there were no large areas of repair in the control group. After treatment in the experimental group, the front, top and back of the condyle were obviously repaired, and the volume of the affected condyle was significantly enlarged. After the data sets were merged, the condyles in the control group exhibited an even distribution of grey and blue, and the repaired bone was not obvious after treatment; however, there were more green segments in the experimental group than in the control group, indicating obvious condylar bone repair (Fig. 4). The CBCT score in the experimental group was significantly lower than that in the control group *(P* < 0.05) at 3 and 6 months after treatment. In the control group, there was no significant change in the CBCT score of the condylar bone before or after treatment (Table 4). The results showed that condylar bone repair occurred in the experimental group and that the reparative effect was significantly better than that in the control group.

**Table 4:** Mann-Whitneyu statistical analysis of CBCT score in two groups of patients with bone disease of temporomandibular joint before and after treatment. Median (P25, P75)

Group	Number	Before treatment	3 months after treatment	6 months after treatment
		Median (P25, P75)	Median (P25, P75)	Median (P25, P75)
Control	30	4.00 (3.75,5.00) 4.00 (4.00,5.00)	4.00 (3.00,5.00)	4.00 (3.00,5.00)
Experimental	30	4.00 (4.00,5.00)	3.00 (2.75,4.00)	3.00 (2.00,3.00)
*P* value		0.747	0.030	0.000

*Note* The control group is SH treatment group and the experimental group is CGF + SH treatment group. (*P* < 0.05)

## Discussion

SH injection into the temporomandibular joint has been widely recognized as one of the simplest and most effective treatment methods. It is a minimally invasive operation, and the relief of joint pain and improvement in the degree of opening can be significantly observed by injecting SH into the subarticular cavity [25, 26]. The intra-articular injection of SH did not destroy the articular cartilage and had little effect on the tissue. Even if SH was repeatedly injected into the articular cavity, only leukocytes, plasma cells and macrophages could be observed in the synovium for a short time. As an exogenous growth factor, CGF can be used as a supplement for the repair of articular cartilage. CGF may play an active role in the molecular response to temporomandibular joint osteoarthritis by mediating the inflammatory response to protect subchondral bone, promoting cell proliferation and differentiation, and inducing tissue repair [27,28]. We chose to combine exogenous SH with CGF because of the quantitative analysis of growth factors at different platelet concentrations by Qiao J et al. [29, 30]. The results showed that CGFs contained more growth factors, such as bone morphogenetic protein (BMP), platelet-derived growth factor (PDGF), insulin-like growth factor (IGF), vascular endothelial growth factor (VEGF), transforming growth factor β1 (TGF-β1) and transforming growth factor β2 (TGF-β2), than other platelet products. These growth factors attract undifferentiated MSCs to sites of trauma. Thus, they promote angiogenesis, chemotaxis and cell proliferation [31], effectively inhibit the expression of inflammatory molecules, reduce the formation of proapoptotic molecules, enhance osteoblast activity, inhibit osteoclast activity, and stimulate tissue regeneration. CGF is an essentially bioactive protein that controls bone and soft tissue regeneration [32]. In addition, CGFs do not quickly lose their effect when acting on joints and can release growth factors continuously for 8–10 days, which is very important in bone healing [33, 34]. Therefore, the key to treating TMJOA is the use of CGF.

Taken together, the findings of the present study, we believe that SH combined with CGF for the treatment of TMJOA can improve clinical symptoms and promote bone repair. This clinical study showed varying degrees of improvement in the symptoms of the 30 patients in the experimental group; for example, the degree of pain, amount of mouth opening, and amount of joint noise decreased significantly, the direction of mandibular movement improved, and condylar cortical bone formation was observed via CT imaging. Although there were differences in the same group before and after treatment, the difference in the experimental group was significantly greater than that in the control group before treatment and at 2 weeks, 3 months and 6 months after treatment, and the therapeutic effect in the experimental group was significantly greater than that in the control group. It was preliminarily confirmed that CGF combined with SH played a positive role in the treatment of TMJOA, and the effect was better than that of SH alone. Specifically, in this study, only SH was used in the control group, and the pain symptoms of the patients were significantly relieved, similar to the results reported by Sun Haibin [35], who described improvements in mouth opening, pain and other clinical symptoms with intra-articular injections of SH in 51 patients with TMJOA. These outcomes are due to the lubrication provided by SH, which reduces joint friction and thus reduces pain; however, the extent to which condylar bone was repaired in patients treated with SH in this study cannot be determined. In our study, after the combination of SH and CGF for the treatment of TMJOA, the degree of joint pain was significantly alleviated, which was reflected in the decrease in the VAS score over time in the experimental group (*P* < 0.05). Dayashankara [36] et al. injected SH into the temporomandibular joint cavity. The VAS score decreased significantly after the operation and at 1 week, 2 weeks, 1 month and 3 months after the operation. In addition, we found that joint dysfunction, mandibular movement disorder, joint and muscle pain and mandibular movement pain were relieved in the experimental group. The Helkimo index in the experimental group was significantly lower than that in the control group(*P* < 0.05). Ahmad [37] et al. reported that the exogenous supplements PRP and SH have similar effects on relieving pain and improving joint function. In the long run, compared with SH, PRP relieves knee joint pain and protects joint function. The effect of reducing dyskinesia is better and lasts longer, which may be related to the platelet dose (volume and concentration) and purity (presence of white blood cells and red blood cells). ShashaL [38] reported that PRP can improve click and joint pain in the temporomandibular joint. These studies are similar to ours and show that exogenous growth factors play a role to some extent in accelerating the recovery of temporomandibular joint function. In addition, through three-dimensional reconstruction, as shown in Fig. 4, condylar changes could be observed more intuitively, accurately and stereoscopically. We observed that the condyle of the control group had no obvious repair at 3 months, and a small amount of repair had occurred at 6 months. After 3 months of treatment, the bone quality of the patients exhibited a certain degree of repair, and at 6 months, there was obvious repair with changes in the volume and shape of the condyle. Sagittal and coronal images (Fig. 3) obtained at 6 months after treatment showed that a small amount of repair also occurred at the top of the condyle in the control group, but there was no significant difference in the CBCT score. The cortical bone in the experimental group healed at 3 and 6 months, with a significantly lower CBCT score than that in the control group. These findings demonstrated that the experimental treatment played a certain role in mitigating the process of condylar bone destruction. In a recent study conducted by Elayah SA [37], 37 patients underwent tooth extraction followed by filling of the alveolar fossa with CGF; postoperative follow-up showed that the soft tissue around the operative area healed faster and better and that the pain was relieved compared with the results in the control group, demonstrating that CGF is a good soft tissue repair material. In addition, Wang [38] used CGF to repair goat condylar bone and showed that CGF plays an important role in the molecular reaction of TMJOA. CGF can mediate inflammation, protect subchondral bone, facilitate cell proliferation, and induce cartilage formation and osteogenesis in TMJOA patients. Several of those studies produced similar results and provided ideas and a basis for our research. In this study, compared with SH alone, the combination of CGF and SH improved the clinical symptoms and bone repair of TMJOA patients better than SH alone, which preliminarily confirmed our conjecture that CGF intervention is an important factor in improving TMJOA.

### Future prospective and limitations

In conclusion, as a therapeutic method, the combination of the two components in the experimental group had synergistic effects on numerous patients, and we expect these combinations to serve as effective methods for the treatment of TMJOA. However, although this clinical study achieved good results, the two drugs were injected directly into the joint cavity. which has several limitations. However, our trials require high-quality, large-scale and long-term clinical follow-up studies. Due to the limited number of participants, neither analysis by age group nor research on the detailed mechanism of the combined application of CGF, including the best mixing ratio, dose, and injection times, could be performed. Furthermore, long-term effects were not observed in this study. It is necessary to perform further basic and clinical experiments to obtain more detailed and accurate information and provide a strong basis for the clinical application of CGF combined with SH in the treatment of TMJOA. If feasible, we hope that this method can be widely used in the clinic and provide a safer and more effective treatment for TMJOA patients. At present, our study mainly focused on the effect of CGF combined with SH on promoting condylar bone remodelling in patients with TMJOA. Clinically, TMJOA patients usually experience pain in the relevant TMJ area, which manifests as temporomandibular joint cavity effusion on MRI. Our further study evaluated the degree of joint pain relief and imaging changes in TMJOA patients with related TMJ effusion before and after treatment with CGF combined with SH via MRI and performed a comprehensive evaluation combined with other clinical indicators.

### Electronic supplementary material

Below is the link to the electronic supplementary material.

Supplementary Material 1

Supplementary Material 2

## Data Availability

The data that support the findings of this study are available from the College of Stomatology, Xi’an Jiaotong University. However, restrictions apply to the availability of these data, which were used under licence for the current study and are not publicly available. However, the data are available from the authors (Xin-yu Jia. Department of General Dentistry and Emergency Room, College of Stomatology, Xi’an Jiaotong University, Xi’an 710004, China. No. 98 Xiwu Road, Xincheng District, Xi’an City, Shaanxi Province. E-mail: 1287066003@qq.com) upon reasonable request and with permission from the authors’ institute.

## References

[1] Kalladka M, Quek S, Heir G, Eliav E, Mupparapu M, Viswanath A. Temporomandibular joint osteoarthritis: diagnosis and long-term conservative management: a topic review. The Journal of Indian Prosthodontic Society. 2014 Mar;14:6–15. 10.1007/s13191-013-0321-3 PMC3935038 24604992

[2] Jiang Q, Qiu YT, Chen MJ (2013). Synovial TGF-beta1 and MMP-3 levels and their correlation with the progression of temporomandibular joint osteoar-thritis combined with disc displacement:apreliminary study. Biomed Rep.

[3] Haghighat Sheila,Oshaghi Samira. Effectiveness of Ozone Injection Therapy in Temporomandibular Disorders.Advanced biomedical research.2020,9. 10.4103/abr.abr_105_20 PMC8012860 33816392

[4] Tanaka E, Detamore MS, Mercuri LG (2008). Degenerative disorders of the tem-poromandibular joint: etiology, diagnosis, and treatment. J Dent Res.

[5] Kopp S, Carlsson GE, Haraldson T, Wenneberg B (1987). Long-term effect of intra-articular injections of sodium hyaluronate and corticosteroid on temporo-mandibular joint arthritis. J Oral Maxillofac Surg.

[6] Cavuoti S, Matarese G, Isola G (2016). Combined orthodontic-surgical man-agement of a transmigrated mandibular canine: a case report. Angle Orthod.

[7] De Riu G, Stimolo M, Meloni SM et al. Arthrocentesis and temporomandib-ular joint disorders: Clinical and radiological results of a prospective study. Int J Dent, 2013; 2013: 790648. 10.1155/2013/790648 PMC3844254 24319462

[8] Aggarwal VR, Lovell K, Peters S et al. Psychosocial interventions for the management of chronic orofacial pain. Cochrane Database Syst Rev.2011: (11): CD008456. 10.1002/14651858.CD008456.pub2 22071849

[9] Januzzi E, Nasri-Heir C, Grossmann E (2013). Combined palliative and anti-inflammatory medications as treatment of temporomandibular joint disc displacement without reduction: a systematic review. Cranio.

[10] Goiato MC, da Silva EV, de Medeiros RA (2016). Are intra-articular injections of hyaluronic acid effective for the treatment of temporomandibular disorders? A sys-tematic review. Int J Oral Maxillofac Surg.

[11] Shi Z, Guo C, Awad M. Hyaluronate for temporomandibular joint disorders. Cochrane Database Syst Rev.1(1), 2003. 10.1002/14651858.CD002970 12535445

[12] Ziegler CM, Wiechnik J, Muhling J (2010). Analysic effects of Intra-articular Morphine in patients with Temporomandibular Joint disorders:a prospective, Double-Blind, placebo-controlled clinical Trial[J]. J Oral Maxillofac Surg.

[13] Daniele M, Marco B, Guarda N. The diagnostic process for temporomandibular disorders[J]. Stomatological, Baltic Dental and Maxillofacial Journal, 2007,286(9):35-39.14.Bjerre Bastos Jonathan J.,Miller Claire P.,Li Yanqi,Andersen Jeppe R.,Karsdal Morten,Bihlet Asger R. Associations between single-question Visual Analogue Scale pain score and weight-bearing and non–weight-bearing domains of Western Ontario and McMaster Universities Arthritis Index pain: data from 2 phase 3 clinical trials[J]. PAIN Reports,2022,7(5). 10.1097/PR9.0000000000001017 PMC9529038 36203646

[14] AlonsoRoyo, Roger. SánchezTorrelo Carmen María,IbáñezVera Alfonso Javier,ZagalazAnula Noelia,CastelloteCaballero Yolanda,ObreroGaitán Esteban,RodríguezAlmagro Daniel,LomasVega Rafael. Validity and Reliability of the Helkimo Clinical Dysfunction Index for the Diagnosis of Temporomandibular Disorders[J]. Diagnostics,2021,11(3). 10.3390/diagnostics11030472 PMC8000811 33800185

[15] Schlueter Brian,Kim Ki Beom,Oliver Donald,Sortiropoulos Gus. Cone beam computed tomography 3D reconstruction of the mandibular condyle.[J]. The Angle orthodontist,2008,78(5). 10.2319/072007-339.1 18298200

[16] Andia I (2014). ABateM. Knee osteoarthritis:hyal-uronic acid,platelet-rich plasma orboth inas-sociation?Expert. Opin Biol Ther.

[17] Musumeci G (2014). Castrogiovanni Paola,Leonardi Rosalia,T.et al. New perspectives for articular cartilage repair treatment through tissue engineering: a contemporary review. World J Orthop.

[18] Sadam Ahmed Elayah. Effect of concentrated growth factor (CGF) on postoperative sequel of completely impacted lower third molar extraction: a randomized controlled clinical study. 2022;22:368. 10.1186/s12903-022-02408-7 PMC9426240 36042448

[19] Trigkilidas D, Anand A (2013). The effectiveness of hyaluronic acid intra-articular injections in managing osteoarthritic knee pain. Ann R Coll Surg Engl.

[20] Nokar Saeid,Sadighpour Leyla,Shirzad Hamed,Shahrokhi Rad Afsaneh,Keshvad Alireza. Evaluation of signs, symptoms, and occlusal factors among patients with temporomandibular disorders according to Helkimo index.Cranio:the journal of craniomandibular practice.2019,37(6). 10.1080/08869634.2018.1449781 29602287

[21] Shi Z, Guo C, Awad M. Hyaluronate for temporomandibular joint disorders. Cochrane Database Syst Rev. 2003; (1): CD002970. 10.1002/14651858.CD002970 12535445

[22] Schlueter B. Kim Ki Beom,Oliver Donald,cone beam computed tomography 3D reconstruction of the mandibular condyle.Angle Orthod.200878(5):880–8. 10.2319/072007-339.1 18298200

[23] Tecco S, Saccucci M, Nucera R (2010). Condylar volume and surface in caucasian young adult subjects. BMC Med Imaging.

[24] Deepak VF. P P.Sodium hyaluronate: an effective adjunct in temporomandibular joint arthrocentesis.[J].Oral and maxillofacial surgery,2016,20(4):405–10. 10.1007/s10006-016-0581-2 27714459

[25] Bertolami A, Gay CN, Clark T. GT, ct al. Use of sodium hyaluronate in treating temporomandibular joint disorders: a randomized, double - blind, placebo - controlled clinical trial [J]. J Oral Maxillofac Surg 1993,51(3):232–42. 10.1016/s0278-2391(10)80163-6 8445463

[26] Xiangjun S, W X A K X H. L.Clinical observation of concentrated growth factor (CGF) combined with iliac cancellous bone and composite bone material graft on postoperative osteogenesis and inflammation in the repair of extensive mandibular defects.[J].Journal of stomatology, oral and maxillofacial surgery,2023,124(6):101472-101472.28.FosterTE,PuskasBL,MandelbaumBRPlatelet-richplasma:From basic science to clinical applications. Am J Sports Med. 37:2259, 2009. 10.1016/j.jormas.2023.101472 37061040

[27] Qiao J, An N, Ouyang X (2017). Quantification of growth factors in different platelet concentrates. Platelets.

[28] Qin J, Wang L, Zheng L (2016). Concentrated growth factor promotes Schwann cell migration partly through the integrin β1-mediated activation of the focal adhesion kinase pathway. Int J Mol Med.

[29] Nma Sac, Suresh S, Sudhakar U (2020). Concentrated growth factor: a effective regenerative tool for soft and hard tissues in periodontics. IP Int J Periodontol Implantol.

[30] Kshirsagar JT, Rubine S (2017). Innovation in regeneration concentrated growth factor. Int J Appl Dent Sci.

[31] Anitua E (1999). Plasma rich in growth factors: preliminary results of use in the preparation of future sites for implants. Int J Oral Maxillofac Implants.

[32] Anitua E, Orive G, Pla R, Roman P, Serrano V, Andía I (2009). The effects of PRGF on bone regeneration and on titanium implant osseointegration in goats: a histologic and histomorphometric study. J Biomed Mater Res Part A.

[33] Sun Haibin,Su Yi,Song Ning,Li.et al. Clinical outcome of Sodium Hyaluronate Injection into the Superior and Inferior Joint Space for Osteoarthritis of the Temporomandibular Joint evaluated by Cone-Beam Computed Tomography: a retrospective study of 51 patients and 56 joints. Medical science monitor:international medical journal of experimental and clinical research.2018,24. 10.12659/MSM.908821 PMC6113854 30122753

[34] Dayashankara KJ, R,Aadya S, ,Rahul K et al. Comparison of efficacy of sodium hyaluronate and normal saline arthrocentesis in the management of internal derangement of temporomandibular joints - a prospective study.[J].National journal of maxillofacial surgery,2019,10(2):217–22. 10.4103/njms.NJMS_26_16 PMC6883885 31798259

[35] Ahmad SR, Parsa H G, Hasan MB (2021). The comparison effects of intra-articular injection of platelet Rich plasma (PRP), plasma Rich in Growth factor (PRGF), Hyaluronic Acid (HA), and ozone in knee osteoarthritis; a one year randomized clinical trial[J]. BMC Musculoskelet Disord.

[36] Sha-Sha L, Li-Li X, ,Li-Kun L (2023). Platelet-rich plasma therapy for temporomandibular joint osteoarthritis: a randomized controlled trial[J]. J Craniomaxillofac Surg.

[37] Elayah Sadam Ahmed,Liang Xiang,Sakran Karim Ahmed.et al.Tu Junbo,na Sijia. Effect ofconcentrated growth factor (CGF) on postoperative sequel of completely impacted lower third molar extraction: a randomized controlledclinical study. BMC Oral Health,2022,22(1). 10.1186/s12903-022-02408-7 PMC9426240 36042448

[38] Feiyu W. Yuhuan Sun,Dongmei He,Lizhen Wang. Effect of concentrated growth factors on the repair of the Goat Temporomandibular Joint. J Oral Maxillofacial Surg 2016,75(3). 10.1016/j.joms.2016.09.006 27725104

