# Supplementary material for: A randomized controlled clinical trial of concentrated growth factor combined with sodium hyaluronate in the treatment of temporomandibular joint osteoarthritis
Source: BMC Oral Health. 2024 May 8;24:540. doi: 10.1186/s12903-024-04258-x (PMC11080079; doi:10.1186/s12903-024-04258-x)
Supplement: Supplementary file 1 — Supplementary Material 1 [file 12903_2024_4258_MOESM1_ESM.docx]

**Supplementary Table 1.** Helkimo index scoring criteria

| Symptom | Criteria | Score |
| --- | --- | --- |
| Impaired range of movement  Impaired TMJ function  Muscle pain  TMJ pain  Pain on movement of the  mandible | Normal range of movement  Slightly impaired mobility  Severe impaired mobility  Smooth movement without  joint sounds and deviation  ≤2 mm  Joint sounds in one or both  joints and deviation ≥2 mm  on opening or closing  Locking or luxation of joint  No tenderness to palpation  Tenderness to palpation in  1–3 sites  Tenderness to palpation in 4  or more sites  No tenderness to palpation  Tenderness to palpation in  1–3 sites  Tenderness to palpation in 4  or more sites  No pain on movements  Pain on 1 movement  Pain on 2 or more movements | 0  1  5  0  1  5  0  1  5  0  1  5  0  1  5 |

**Supplementary Table 2.**Condylar imaging score

| Scoring items |  | Scoring standard | |  |
| --- | --- | --- | --- | --- |
|  | 0 | 1 | 2 | 3 |
| Flat | Nnoe | Slight | Moderate | Extensive |
| Bone destruction | Nnoe | Cortical bone | Subcortical layer | Cancellous bone |
| Osteophytes | Nnoe | <1mm | 1～2mm | >2mm |
| Sclerosis | Nnoe | <1/2 | ≥1/2 |  |
| Cystic degeneration | Nnoe | Small | Larger or more |  |
