# Supplementary material for: A randomized controlled clinical trial of concentrated growth factor combined with sodium hyaluronate in the treatment of temporomandibular joint osteoarthritis
Source: BMC Oral Health. 2024 May 8;24:540. doi: 10.1186/s12903-024-04258-x (PMC11080079; doi:10.1186/s12903-024-04258-x)
Supplement: Supplementary file 2 — Supplementary Material 2 [file 12903_2024_4258_MOESM2_ESM.docx]

**table 1** | CONSORT 2010 checklist of information to include when reporting a randomised trial*

| Section/Topic | Item No | Checklist item |
| --- | --- | --- |
| Title and abstract |  |  |
|  | 1a | Identification as a randomised trial in the title |
|  | 1b | Structured summary of trial design, methods, results, and conclusions (for specific guidance see CONSORT for abstracts |
| Introduction |  |  |
| Background and objectives | 2a | Scientific background and explanation of rationale |
|  | 2b | Specific objectives or hypotheses |
| Methods |  |  |
| Trial design | 3a | Description of trial design (such as parallel, factorial) including allocation ratio |
|  | 3b | Important changes to methods after trial commencement (such as eligibility criteria), with reasons |
| Participants | 4a | Eligibility criteria for participants |
|  | 4b | Settings and locations where the data were collected |
| Interventions | 5 | The interventions for each group with sufficient details to allow replication, including how and when they were actually administered |
| Outcomes | 6a | Completely defined pre-specified primary and secondary outcome measures, including how and when they were assessed |
|  | 6b | Any changes to trial outcomes after the trial commenced, with reasons |
| Sample size | 7a | How sample size was determined |
|  | 7b | When applicable, explanation of any interim analyses and stopping guidelines |
| Randomisation: |  |  |
| Sequence generation | 8a | Method used to generate the random allocation sequence |
|  | 8b | Type of randomisation; details of any restriction (such as blocking and block size) |
| Allocation concealment mechanism | 9 | Mechanism used to implement the random allocation sequence (such as sequentially numbered containers),describing any steps taken to conceal the sequence until interventions were assigned |
| Implementation | 10 | Who generated the random allocation sequence, who enrolled participants, and who assigned participants to interventions |
| Blinding | 11a | If done, who was blinded after assignment to interventions (for example, participants, care providers, those assessing outcomes) and how |
|  | 11b | If relevant, description of the similarity of interventions |
| Statistical methods | 12a | Statistical methods used to compare groups for primary and secondary outcomes |
|  | 12b | Methods for additional analyses, such as subgroup analyses and adjusted analyses |
| Results |  |  |
| Participant flow (a diagram is strongly recommended) | 13a | For each group, the numbers of participants who were randomly assigned, received intended treatment, and were analysed for the primary outcome |
|  | 13b | For each group, losses and exclusions after randomisation, together with reasons |
| Recruitment | 14a | Dates defining the periods of recruitment and follow-up |
|  | 14b | Why the trial ended or was stopped |
| Baseline data | 15 | A table showing baseline demographic and clinical characteristics for each group |
| Numbers analysed | 16 | For each group, number of participants (denominator) included in each analysis and whether the analysis was by original assigned groups |
| Outcomes and estimation | 17a | For each primary and secondary outcome, results for each group, and the estimated effect size and its precision (such as 95% confidence interval) |
|  | 17b | For binary outcomes, presentation of both absolute and relative effect sizes is recommended |
| Ancillary analyses | 18 | Results of any other analyses performed, including subgroup analyses and adjusted analyses, distinguishing prespecified from exploratory |
| Harms | 19 | All important harms or unintended effects in each group |
| Discussion |  |  |
| Limitations | 20 | Trial limitations, addressing sources of potential bias, imprecision, and, if relevant, multiplicity of analyses |
| Generalisability | 21 | Generalisability (external validity, applicability) of the trial findings |
| Interpretation | 22 | Interpretation consistent with results, balancing benefits and harms, and considering other relevant evidence |
| Other information |  |  |
| Registration | 23 | Registration number and name of trial registry |
| Protocol | 24 | Where the full trial protocol can be accessed, if available |
| Funding | 25 | Sources of funding and other support (such as supply of drugs), role of funders |

David M ,Sally H ,F K S , et al.CONSORT 2010 explanation and elaboration: updated guidelines for reporting parallel group randomised trials.[J].BMJ (Clinical research ed.),2010,340(8):c869.

**1a:** A clinical randomized controlled trial of concentrated growth factors combined with sodium hyaluronate in the treatment of temporomandibular joint osteoarthritis。

**1b: Objective:** To investigate the effect of concentrated growth factor (CGF) combined with sodium hyaluronate (SH) on temporomandibular joint osteoarthritis (TMJOA).

**Methods:** Sixty patients with TMJOA who were diagnosed by cone-beam computed tomography (CBCT) from March 2020 to March 2023 at the Stomatological Hospital of Xi'an Jiaotong University were randomly divided into a control group (n = 30) and an experimental group (n = 30). The patients in the experimental group were treated with CGF + SH, and those in the control group were treated with SH only. The visual analogue scale (VAS) score of pain in the temporomandibular joint (TMJ)area, the Helkimo Clinical Dysfunction Index (Di) and the changes on condylar CBCT imaging at the first visit and 2 weeks, 3 months and 6 months after treatment were recorded. The CBCT data of the patients in the experimental and control groups were collected, the three-dimensional CBCT image sequences were imported into Mimics Medical 19.0 software in DICOM format for condylar reconstruction.

**Results:** The VAS score at 2 weeks, 3 months and 6 months after treatment were significantly lower in the experimental group than in the control group (*P* < 0.05), and the pain in the experimental group was significantly relieved. The Di was significantly lower in the experimental group than in the control group (*P* < 0.05), and the clinical function of the TMJ was improved. After treatment, the CBCT score was significantly lower in the experimental group than in the control group (*P* < 0.05), and the condylar bone cortex was obviously repaired. Observation of the condylar bone cortex by three-dimensional reconstruction showed the same results as CBCT.

**2a:** Temporomandibular joint osteoarthritis (TMJOA) is a chronic degenerative disease that occurs in the TMJ area under the action of many factors. It is the most serious type of TMJ disorder [1], usually leading to destruction of the mandibular condyle and articular fossa due to the increase in joint load. TMJOA can cause varying degrees of clinical symptoms, including joint pain, clicking, limited movement and limited mouth opening, resulting in loss of joint function [2]. At present, conservative treatments, most commonly intra-articular injections, are the main treatment strategies applied in the clinic. Many kinds of drugs are used in these treatments, including hormones, ozone and sodium hyaluronate (SH). Among them, hormones can relieve pain and increase the range of motion of the joint in the short term, but they can cause damage to the cartilage and bone structure in the articular cavity. Ozone causes strong oxidation, which can promote cell metabolism and enhance the ability for cell repair, but the effect lasts for a short time [3]. These treatments can improve the symptoms of TMJOA, but there is no gold standard for the treatment of TMJOA [4-9].

Sodium hyaluronate (SH) is the main component of synovial fluid, which can reduce friction caused by joint motion, lubricate joints, improve physiological joint function, and protect joints through anti-inflammatory mechanisms [10,11]. SH is the most commonly used drug for intra-articular injections in the treatment of TMJOA. Injection of sodium hyaluronate into the upper cavity of the temporomandibular joint is more effective than the subarticular cavity in the treatment of synovitis of the joint. The possible reason is that compared with the subarticular cavity, the volume of the upper cavity is larger and the injection is relatively easy[12]. However, it has been proved that the subarticular cavity surface is rougher than the upper cavity surface, and it is often prone to condylar surface fibrosis and degeneration. Subarticular cavity injection can significantly improve the clinical effect of TMD patients with poor effect of upper cavity injection^[13]^.SH has been widely used in the TMJ and other large joints. For example, the clinical efficacy and safety of intra-articular injections of SH in the knee joint, ankle joint and hip joint have been systematically reviewed and supported by clinical studies, but most of the previously published articles have focused on changes in clinical signs and symptoms after intra-articular injections of SH [12-16]. There are no clear results showing that SH can mitigate bone destruction.

Platelet-rich plasma (PRP), which comes from autologous sources, rarely produces an immune rejection reaction. Regimens of SH combined with PRP have been used in the clinical treatment of TMJOA [17,18], but PRP carries some potential immunogenic risk and has a short action time. Additionally, its effect on hard tissue recovery is not clear. Thus, new replacements are urgently needed in the clinic. Autologous concentrated growth factor (CGF) contains more growth factors, has a stronger ability to support tissue regeneration and repair, can better promote the regeneration of bone, blood vessels, fibres and other tissues, and has higher biosafety. CGF can be used to reconstruct damaged bone and cartilage, achieve intra-articular homeostasis, and regulate inflammation [19]. At present, CGF has been used in the treatment of OA in all parts of the body, but there have been few reports on the application of CGF in the TMJ. In this study, CGF was used as an endogenous growth factor source in patients randomly divided into groups to analyse the advantages of CGF combined with SH in the treatment of TMJOA. Through various scoring standards, this work proposes a new theoretical basis for the clinical treatment of TMJOA.

**2b:** In this study, CGF was used as an endogenous growth factor source in patients randomly divided into groups to analyse the advantages of CGF combined with SH in the treatment of TMJOA. Through various scoring standards, this work proposes a new theoretical basis for the clinical treatment of TMJOA.

3a: control group: SH ; experimental group: SH+ CGF

3b: A total of 62 patients agreed to participate in clinical studies when they were treated for TMJOA. Inform all patients of the treatment in the trial. The age of the patients ranged from 18 to 50 years old. 2 patients were not suitable for this test because of diabetes. Therefore, a totle of 60 patients were enrolled in the experiment, including 30 females and 30 males.

**4a:** The inclusion criteria were as follows: (1) diagnosis of TMJOA made according to patient history, clinical symptoms, joint function and imaging examination and treatment by the same physician at the Stomatology Hospital of Xi'an Jiaotong University, the injection was performed by the same doctor;(2) voluntary agreement to participate in the study and provision of a signed informed consent form; (3) good overall condition, without serious systemic diseases or mental health conditions.

**4b:** Sixty TMJOA patients were recruited from the Department of Oral and Maxillofacial Surgery, College of Stomatology, Xi'an Jiaotong University

**5:** Before the injection, the patient was informed the treatment plan and after the injection, the follow-up time was at the after injection in 2 weeks, 3 months, and 6 months, the experiment could be terminated at any time if the patient requests withdrawal or adverse complications was occurred. In the SH treatment group, the patient assumed a sitting position , the 1cm in front of the tragus and the line between the lateral canthus and the tragus are marked by about 2mm. the patient was instructed to open the mouth repeatedly, while the doctor touched the condyle on the affected side. The doctor marked the needle entry point outside the condyle, 0.5%povidone iodine disinfected the operation area with the mouth closed, inserted the needle vertically with respect to the outer pole of the condyle, and instructed the patient to open the mouth a small amount. At this time, the needle can move with the condyle; if this occurred, the doctor slid the injection needle tip upwards and back to the posterior slope of the condyle and then injected 1 ml of lidocaine if there was no resistance, withdrawing some fluid to confirm that the needle entered the subarticular cavity. After repeatedly washing the joint cavity, the flushing fluid was withdrawn, and 1 ml of SH was injected. After the injection, the patient was instructed to open and close the mouth repeatedly to evenly distribute the drug in the joint cavity. All patients were instructed to avoid opening their mouths widely, pay attention to avoid biting hard objects in their diet, and rest and keep warm. In the experimental group, 0.5 ml of CGF combined with 0.5 ml of SH was injected. In the control group, 1 ml of SH was injected, and the operation was the same as that in the experimental group.

**6a:** Primary outcome: condylar bone repair, secondary outcome: clinical symptoms improvement. Evaluation time: before injection, 2 weeks, 3 months, 6 months after injection

**6b:** Indicator not changed.With reference to the previous literature, we believe that the success rate of the hypothetical target is high, and the experimental results also confirm this.

**7a:** The sample size was calculated according to the completely random design sample size estimation formula.

**7b:** Did not stop, unified data analysis was carried out after the end of the experiment

**8a:** Write the control group (SH) or the experimental group (SH + CGF) in an opaque, sealed envelope. The envelope was randomly and equally distributed to patients

**8b:** Completely randomized grouping

**9:** The envelope was given to doctor A to injection it. Doctor B conducted clinical analysis and collected data. The grouping results were announced 6 months after treatment.

**10:** People who generate random assignment sequences and assign sequences to subjects do not participate in the experiment.

**11a:** Single blind, the patient doesn't know.

**12a:** Primary indicator：the non-normal distribution data was expressed by quartile (25th percentile, 75th percentile), and mann-whitneyu test was used for statistics. The difference was statistically significant secondary indicator：the normal distribution data was expressed by average ±standard deviation (‾x±s ), the t-test was used for data statistics

**13a,13b:** The number of 30 cases was randomly assigned to each group. See flowchart

**14a:** March 2020 and March 2023.

**14b:** uninterrupted

**15:**Table1.

**16:**man:30.women:30.

**17a:** Main index results: The experimental group was significantly repaired compared with the control group, with statistical significance

Results of secondary indexes: The clinical symptoms of the experimental group were improved compared with the control group, which had statistical significance

**18:** Pre-set analysis: Bone repair, mouth opening, muscle pain, joint pain, dysfunction, jaw movement were all improved

Unconfigured analysis: None

**19:**None

**20:** In conclusion, as a therapeutic method, the hybrid of the two components in the experimental group showed a positive effect, and we expect their hybrid to serve as an effective method for the treatment of TMJOA. However, although this clinical study has achieved good results, the two drugs were injected directly into the joint cavity. which has some limitations. Our trials require high-quality, large-scale and long-term clinical follow-up studies. In our experiment, due to the limited number of participants, neither analysis by age group nor research on the detailed mechanism of the combined application of CGF and , including the best mixing ratio, dose, and injection times, could be performed. Furthermore, the long-term effects were not observed in this study. It is necessary to perform further basic and clinical experiments to obtain more detailed and accurate information and provide a strong basis for the clinical application of CGF combined with SH in the treatment of TMJOA.

**21:** CGF is simple and easy to obtain, and has low rejection.If feasible, we hope that this method can be more widely used in clinic, and provide a more safe and effective treatment for TMJOA patients.

**22:** In this study, the hybrid of CGF and SH can better improve the clinical symptoms and bone repair of TMJOA patients compared with SH only, which preliminarily confirms our conjecture that the intervention of CGF is an important factor in the improvement of TMJOA.

**23:** Registration number and name of trial registry：ChiCTR2400082712.

Subject:A clinical randomized controlled trial of concentrated growth factors combined with sodium hyaluronate in the treatment of temporomandibular joint osteoarthritis

**24:**Clinical Trial Management PublicPlatform http://www.medresman.org.cn/login.aspx.

**25:** College of Stomatology, Xi'an Jiaotong University: pharmacy
